# A study of sex and age related heterogeneity in thyroid-gonadal axis interactions in a multi-center cohort of individuals with normal thyroid function

**DOI:** 10.3389/fendo.2026.1848966

**Published:** 2026-05-29

**Authors:** Lina Yang, Haoran Hu, Qunhao Li, Jianjun Yang, Huxiao Liu, Mengya Chen

**Affiliations:** 1 Development Department of the Wisdom Hospital, Shandong Provincial Third Hospital, Shandong University, Jinan, Shandong, China; 2 Shandong Provincial Third Hospital, Shandong University, Jinan, Shandong, China; 3 Key Laboratory of Endocrine Glucose and Lipids Metabolism and Brain Aging, Ministry of Education, Department of Endocrinology, Shandong Provincial Hospital Affiliated to Shandong First Medical University, Jinan, Shandong, China; 4 Department of General Practice, Shandong Provincial Third Hospital, Shandong University, Jinan, Shandong, China; 5 Endocrinology Department, Jining Second Hospital, Jining, Shandong, China; 6 Department of the Medical Records, Shandong Provincial Third Hospital, Shandong University, Jinan, Shandong, China

**Keywords:** age-dependent heterogeneity, euthyroid population, sex differences, sex hormones, thyroid hormones

## Abstract

**Introduction:**

There is a complex physiological interaction between the HPT axis and the HPG axis. However, previous studies have primarily focused on individuals with thyroid dysfunction, and our understanding of the relationship between these two axes in individuals with normal thyroid function remains limited.

**Methods:**

This study based on multi-center cross-sectional data, included 1,025 healthy participants with no known endocrine disorders. It systematically assessed the association between thyroid hormones and sex hormones, with a focus on analyzing effects stratified by gender and age.

**Results:**

The results showed that FT4 was positively correlated with T in men (Std β = 0.448, P = 0.002), while FT3 and TSH did not show a significant effect. No significant association was observed in the overall female population, but a clear trend emerged when the data were stratified by age. In the <18-year-old group, thyroid hormones and gonadotropins showed a negative correlation (e.g., FT3–LH: r = −0.372, P < 0.001). In the 18–40-year-old group, a positive correlation was observed (FT4–FSH: r = 0.125, P = 0.001), whereas this correlation disappeared in the >40-year-old group. In women of childbearing age, multivariate analysis further confirmed the association between FT4 and FSH (Std β = 0.118, P = 0.005) and between TSH and LH (Std β = 0.136, P < 0.001).

**Discussion:**

The study findings indicate that the association between thyroid hormones and sex hormones exhibits significant gender differences and is age-dependent. Among women, the direction of this association varies. This suggests that pooled analyses may mask the true association due to population heterogeneity, thereby underestimating the actual association. This study reveals the potential role of normal fluctuations in thyroid hormone levels in the regulation of reproductive endocrinology and provides new epidemiological evidence for research into the mechanisms underlying related reproductive endocrine disorders and for the early identification of risks.

## 1 Introduction

Within the complex neuroendocrine regulatory network, the hypothalamic-pituitary-thyroid (HPT) axis and the hypothalamic-pituitary-gonadal (HPG) axis are not independent of one another but are closely interconnected. Previous studies have shown that, in addition to playing a role in energy metabolism and the maintenance of internal homeostasis, thyroid hormones are also closely associated with the development of reproductive organs, the maintenance of gonadal function, and gametogenesis (1, 2). From an anatomical and molecular perspective, thyroid hormone receptors and related signaling pathways are present not only at the central regulatory level but are also distributed in peripheral reproductive target organs such as the testes, ovaries, and endometrium (3, 4). Clinically, this inter-axial relationship is more pronounced in cases of thyroid dysfunction. Hyperthyroidism or hypothyroidism is often accompanied by reproductive endocrine abnormalities, such as abnormal semen parameters, ovulatory dysfunction and changes in endometrial receptivity (5). These findings suggest that there may be a continuous and complex physiological interaction between the HPT axis and the HPG axis.

However, current understanding of the interaction between these two axes still stems primarily from studies of pathological conditions such as thyroid disease, gonadal dysfunction, or polycystic ovary syndrome (6, 7). In contrast, there is currently limited evidence to suggest whether physiological fluctuations in thyroid hormone levels within the reference range are still associated with sex hormone levels in individuals with normal thyroid function. In particular, there has been no systematic evaluation of whether this association exists independently, or whether its direction and strength are consistent, especially among individuals with no clear evidence of thyroid or pituitary disease. Since previous studies have largely been based on specific disease cohorts or observational data with small sample sizes, the relationship between thyroid hormone variability within the normal range and reproductive endocrine markers remains poorly understood.

Furthermore, research on this issue is also hampered by significant population heterogeneity. First, there are differences between men and women in terms of sex hormone secretion levels, feedback regulation mechanisms, and the maintenance of endocrine homeostasis (8, 9). Failure to make distinctions in the analysis may result in gender-related association patterns being confounded (10). Second, the female reproductive endocrine system undergoes distinct phase-specific changes during puberty, the reproductive years, and the perimenopausal period (11). The relationship between thyroid hormones and gonadotropins may not remain constant across different life stages and may even exhibit inconsistent trends (12). Therefore, conducting a gender-stratified analysis and further stratifying women by age can help identify potential differential patterns of association between the thyroid-gonadal axis more accurately.

Against this backdrop, this study utilized a large, multi-center clinical cohort and strictly excluded participants with thyroid disease, pituitary disorders, and other underlying conditions that could affect endocrine function. Using a cross-sectional study design to explore the association between thyroid hormones and sex hormones. The primary objectives of this study are: First, to describe the patterns of correlation between thyroid hormones and sex hormones in the general population and across different genders. Second, to further evaluate the adjusted association between relevant thyroid markers and androgen levels in men. Third, we conducted an age-stratified analysis among women to examine the patterns of change in the relationship between thyroid hormones and gonadotropins across different life stages. Based on the above analysis, this study aims to provide clinical and epidemiological insights into the potential relationship between the HPT axis and the HPG axis in individuals with normal thyroid function.

## 2 Data and methods

### 2.1 Research design and research subjects

This study employed a multi-center cross-sectional design. The data were obtained from the electronic medical records and clinical laboratory databases of Shandong Provincial Third Hospital and Jining Second Hospital. Data was collected from January 2022 to January 2026. The study population consists of individuals who underwent both thyroid hormone and sex hormone testing during the same medical visit. To avoid duplicate inclusion, if a subject has multiple test records, only the first record in which both types of hormone tests were completed simultaneously will be included in the analysis.

The aim of this study is to evaluate the relationship between thyroid hormones and sex hormones in individuals with normal thyroid function. To avoid interference with the endocrine system from other factors, this study established strict inclusion and exclusion criteria. Exclusion criteria specifically include: (1) Individuals with a past or current confirmed diagnosis of primary or secondary thyroid dysfunction (such as hyperthyroidism, hypothyroidism, autoimmune thyroiditis, etc.). (2) Patients with a radiological or pathological diagnosis of a pituitary tumor, a large adenoma, or other organic lesions of the central nervous system that clearly affect central endocrine feedback pathways. (3) Individuals with severe hepatic or renal insufficiency, malignant tumors, or other systemic diseases that may cause systemic endocrine or metabolic disorders. (4) Individuals who have undergone exogenous hormone replacement therapy, used ovulation-inducing medications, or taken drugs known to interfere with thyroid or gonadal function within the past 3 months. (5) Samples with significant gaps in test data. (6) Exclude patients who are pregnant. Following the rigorous data cleaning and screening process described above, a core analysis cohort comprising 1,025 eligible participants was ultimately established.

In this study, “normal thyroid function” is defined as: thyroid-stimulating hormone(TSH), free triiodothyronine(FT3), and serum levels of free(FT4)levels in all subjects falling within the respective reference ranges of the participating centers’ laboratories, and no prior documented diagnosis of hyperthyroidism, hypothyroidism, or other specific thyroid disorders.

### 2.2 Laboratory parameters and variable definitions

Thyroid function typically involves measuring serum levels of free (FT4, pmol/L, reference value:12--22), free triiodothyronine (FT3, pmol/L, reference value: 3.1-6.8), and thyroid-stimulating hormone (TSH, μIU/mL, reference value:0.27--4.2). The sex hormone panel includes testosterone (T, ng/mL, reference value:0.029--0.481), estradiol (E2, pg/mL, reference value:12.4--233), progesterone (P, nmol/L, reference value:0.181--2.84, Menopause:0.159-0.401), luteinizing hormone (LH, mIU/mL, reference value:12.4-341, menopause: 0.159-0.401), and follicle-stimulating hormone (FSH, mIU/mL, reference value: 1.5-21.5; menopause: 25.8-134.8). Laboratory tests for patients are performed on an empty stomach. All data were derived from routine laboratory test results at the two hospitals. Both centers used the same testing platforms and reference ranges.

At the same time, based on the raw measurement values, we further calculated the FT3/FT4 ratio, which reflects peripheral deiodination efficiency, as well as the thyroid feedback quantile-based index (TFQI), which is used to assess central thyroid hormone feedback sensitivity (13). The formula for TFQI is:


TFQI=cdf(FT4)−[1−cdf(TSH)]

cdf stands for cumulative distribution function. TFQI is used to assess central feedback sensitivity in the HPT axis; a higher value indicates a relative decrease in central thyroid hormone sensitivity.

Because this study employed a multicenter design, data from the Third Hospital of Shandong Province and Jining Second Hospital were compared prior to analysis. There were no significant differences in baseline characteristics or primary clinical outcomes among the centers; therefore, center was not included as a covariate in the multivariate regression model. Future studies may further evaluate potential center-specific effects using mixed-effects models or fixed-effects center analysis.

### 2.3 Stratification strategies and study outcomes

Given that there are gender differences and that women’s reproductive endocrine status is highly age-dependent, this study first conducted a stratified analysis by gender. Among women, the population was further divided into three subgroups based on age: <18 years, 18–40 years, and >40 years, to approximate the physiological differences characteristic of puberty, childbearing age, and the pre- and post-menopausal periods. <18, the HPG axis is active and in a developmental phase; between the ages of 18 and 40, hormonal homeostasis is relatively stable; and after age 40, FSH and LH levels rise while sex hormone secretion declines (12). This stratification roughly reflects the state of the HPG axis during different physiological stages in women and helps identify age-dependent differences in the relationship between thyroid hormones and sex hormones. It should be noted that this age stratification serves only as a rough approximation of the different life stages in women and cannot replace more precise stratification based on the menstrual cycle, pregnancy status, or menopausal status.

This study primarily focuses on the relationship between thyroid function markers and key sex hormone markers. The study focuses on evaluating the association between thyroid hormones and T. In women, the focus is on evaluating the patterns of association between thyroid hormones and LH, FSH and P. For indicators that showed a statistically significant association in the univariate analysis, we further constructed a multivariate linear regression model to validate the findings.

### 2.4 Statistical analysis

(1) Descriptive statistics and tests of significance: All continuous variables underwent a Shapiro-Wilk normality test prior to analysis to determine whether the data followed a normal distribution. For continuous data that follow a normal distribution, the mean ± standard deviation ( $\bar{x}$ ± SD) is used; independent samples t-tests are used for comparisons between groups. For non-normally distributed quantitative data, the median was used as the measure of central tendency, and the Mann-Whitney U test was used for comparisons between groups. For men, FT4 and TFQI are expressed as $\bar{x}$ ± SD; all other variables are expressed as medians.

(2) Correlation analysis: To illustrate the trends in hormone levels over time, age-related trajectory plots were generated using locally weighted regression smoothing (LOESS) (14) and presented by gender. At the same time, to assess the statistical associations between thyroid function markers and sex hormones and their derivatives, this study employed Spearman’s rank correlation analysis, conducting separate analyses for each gender group to examine the univariate associations between thyroid hormones and sex hormones. Use Spearman’s rank correlation analysis to calculate the correlation matrix between thyroid markers and sex hormone markers, along with the corresponding P-value matrix. In the analysis, missing values were handled using `nan_policy=‘omit’. To present the results visually, a heatmap is used to display the full matrix of correlation coefficients, with color intensity representing the magnitude of the correlation coefficients, and the values labeled on the map. All analyses and visualizations were performed using the pandas, numpy, scipy, matplotlib, and seaborn packages in Python 3.9, with a significance level set at P < 0.05 (15).

(3) Multivariate regression analysis: In multivariate analysis, a multiple linear regression model was constructed using the variables identified as having an association in the univariate analysis (16). The male model uses T as the dependent variable. The female reproductive-age models use LH, FSH, and P as dependent variables. The independent variables include FT3, FT4, TSH and age. To compare the strength of the effects of each variable, the report presents both unstandardized regression coefficients (β) and standardized regression coefficients (Std β). Prior to modeling, multicollinearity was assessed using the variance inflation factor (VIF) (17). A VIF value of less than 5 was considered to indicate no significant multicollinearity. All statistical tests were considered statistically significant at P < 0.05. In the multivariate regression analysis of the male cohort, triglyceride (TG) levels were included in addition to age and thyroid function markers to partially adjust for confounding by metabolic factors. In the female cohort, because BMI and menstrual cycle data were not collected and the missing rate for metabolic indicators exceeded 80%, these factors could not be included in the multivariate regression analysis; therefore, residual confounding may be present.

## 3 Results

### 3.1 Baseline characteristics of the study population

After screening, a total of 1,025 subjects were ultimately included in the statistical analysis, including 52 males and 973 females. The median age of the overall study population was 31. Compared with men, women had significantly higher baseline age (31vs 26.5, P = 0.007). **Table 1** for the detailed distribution of the baseline data.

**Table 1:** Statistical analysis of baseline and biochemical characteristics.

Variable	Total	Male	Women	P value
age	31.00 (24.00-40.00)	26.50 (14.75-38.50)	31.00 (25.00-40.00)	0.0071
TSH	2.02 (1.40-2.87)	1.91 (1.35-2.50)	2.03 (1.41-2.90)	0.1524
FT3	4.86 (4.39-5.36)	5.55 (5.06-6.64)	4.83 (4.38-5.29)	0
FT4	16.10 (14.60-17.70)	16.72 ± 3.65	16.10 (14.60-17.60)	0.0482
LH	6.84 (4.15-12.19)	3.87 (2.20-5.96)	7.09 (4.31-12.48)	0
FSH	6.07 (4.69-7.79)	4.02 (2.88-5.21)	6.14 (4.78-7.91)	0
E2	43.58 (26.49-75.05)	26.53 (13.21-39.52)	44.55 (27.05-77.59)	0
P	0.85 (0.56-1.46)	0.64 (0.46-0.91)	0.88 (0.57-1.49)	0.0001
T	0.34 (0.22-0.50)	3.46 (0.53-5.60)	0.33 (0.22-0.48)	0
FT3/FT4	0.30 (0.27-0.34)	0.34 (0.27-0.44)	0.30 (0.27-0.34)	0.0003
LH/FSH	1.04 (0.63-1.92)	0.87 (0.58-1.22)	1.05 (0.63-1.97)	0.019
E2/P	44.02 (18.59-87.39)	38.48 (24.28-60.57)	44.63 (18.41-89.17)	0.1818
LH/T	20.54 (12.72-34.62)	1.13 (0.73-2.37)	21.07 (13.60-35.17)	0
TFQI	0.00 (-0.27-0.27)	0.03 ± 0.42	-0.00 (-0.27-0.26)	0.1895

Normally distributed continuous variables were depicted by Mean ± SD, and nonnormally distributed continuous variables were represented as Q2 (Q1, Q3). Q1: First quartile; Q2: Second quartile; Q3: Third quartile. TSH: thyroid-stimulating hormone; FT3: free triiodothyronine; FT4: serum levels of free; LH: luteinizing hormone; FSH: follicle-stimulating hormone; E2: estradiol; P: progesterone; T: testosterone;.

With regard to thyroid function markers, male participants had higher levels of FT3 (5.55 vs. 4.83, P < 0.001) and FT4 (P = 0.048) than female participants. The FT3/FT4 ratio was also higher in the male population (P < 0.001). However, there were no statistically significant differences in the TSH and TFQI indices between the two groups (P > 0.05). With regard to sex hormone levels, testosterone levels in the male cohort were significantly higher than those in the female cohort (3.46 vs. 0.33, P < 0.001). Levels of LH, FSH, E2, and P were all higher in women than in men (P < 0.001). With regard to hormone ratios, women had significantly higher LH/FSH and LH/T ratios than men (P = 0.019 and P < 0.001), but no significant difference was observed in the E2/P ratio between the two groups (P = 0.182).

### 3.2 Trends in hormone levels with age

**Figure 1:**
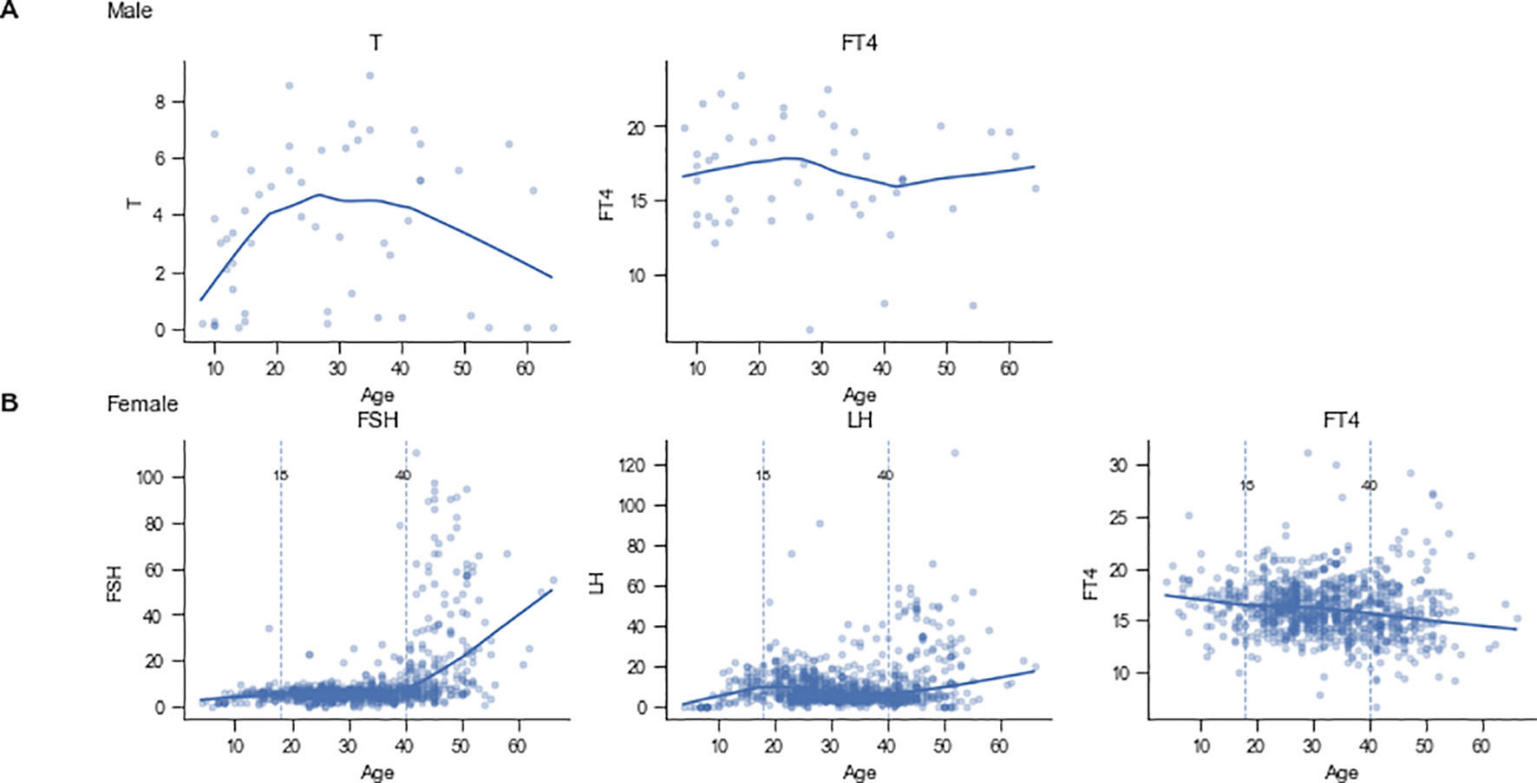
Age-dependent hormonal trajectories. **(A)** shows T and FT4 levels in men. **(B)** shows FSH, LH, and FT4 levels in women.

LOESS smoothing curves were used to visualize the trends in major thyroid hormones and sex hormones over time. The results are shown in **Figure 1**.

In men, T levels follow a nonlinear trend with age, rising in early adulthood and then gradually declining as men age. In contrast, FT4 shows relatively little overall variation across different age groups. These findings suggest that, within the normal range of thyroid function, age-related changes in androgen levels are more pronounced than those in thyroid hormones.

In women, gonadotropin levels exhibit more pronounced age-related changes. In particular, FSH levels remain relatively stable during early adulthood but rise significantly after the age of 40. LH levels also tend to rise with age, but the increase is less pronounced than that of FSH. In comparison, FT4 levels show less variation across different age groups in women. These trends suggest that female sex hormones fluctuate more significantly with age, while thyroid hormone levels remain relatively stable overall.

### 3.3 Characteristics of the relationship between thyroid and sex hormones in the general population

**Figure 2:**
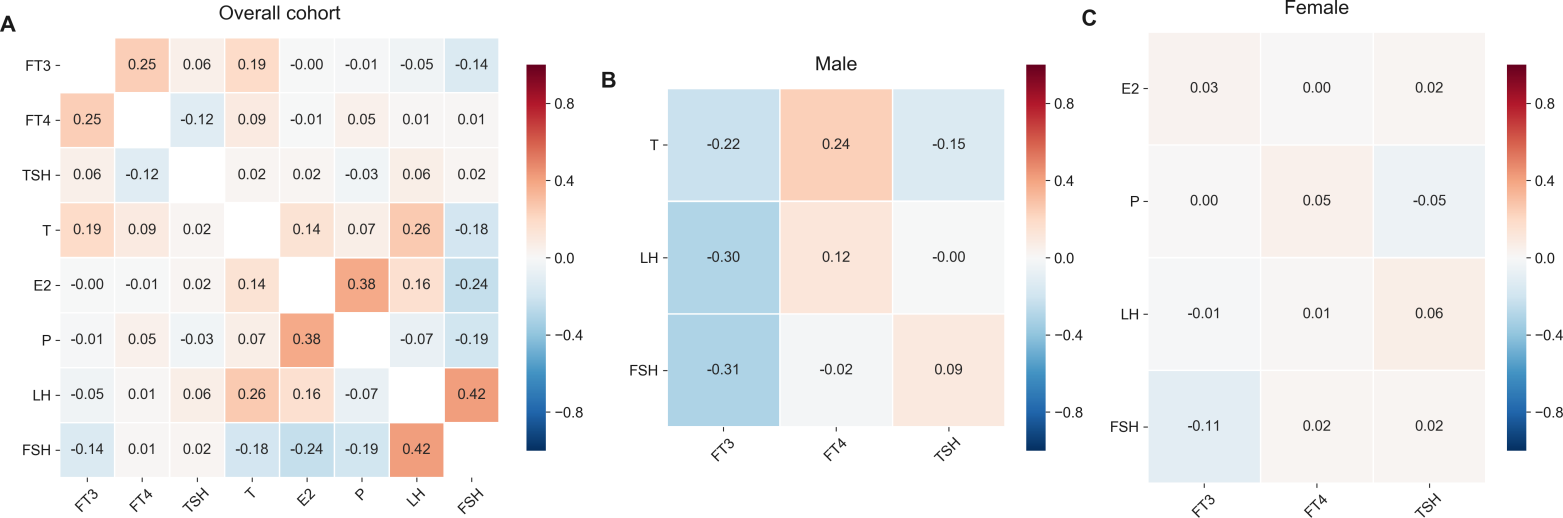
Correlation analysis; **(A)** Correlation in the overall population; **(B)** Correlation in males; **(C)** Correlation in females.

In the overall study population, Spearman’s correlation analysis revealed a generally weak correlation between thyroid hormones and sex hormones (**Figure 2A**). In particular, FT3 showed a weak positive correlation with T (r = 0.189, P < 0.001). In addition, there was a significant positive correlation between FT3 and FT4 within the thyroid hormone group (r = 0.246, P < 0.001). Among the sex hormones, significant positive correlations were observed between LH and FSH (r = 0.419), E2 and P (r = 0.376), T and LH (r = 0.261) (all P < 0.001).

After stratification by sex, FT3 was negatively correlated with both LH and FSH in men (LH: r = −0.302, P = 0.030; FSH: r = −0.306, P = 0.027)(**Figure 2B**). In addition, a positive correlation was observed between FT4 and T, although it did not reach statistical significance (r = 0.242, P = 0.085). No statistically significant correlations were observed between TSH and the various sex hormone levels (all P > 0.05). In the overall correlation analysis of the female population (**Figure 2C**), no significant correlations were observed between FT3, FT4, and TSH and any of the sex hormones (all P > 0.05).

Univariate correlation analysis may be confounded by potential covariates such as age. Therefore, to further verify the independence of these associations, this study subsequently constructed a multivariate regression model incorporating the age variable in the male cohort. For women, given that the female reproductive endocrine system is highly dependent on age-related changes, research should be conducted by stratifying women into specific age groups.

### 3.4 Multivariate regression analysis of thyroid hormones and sex hormones in men

**Figure 3:**
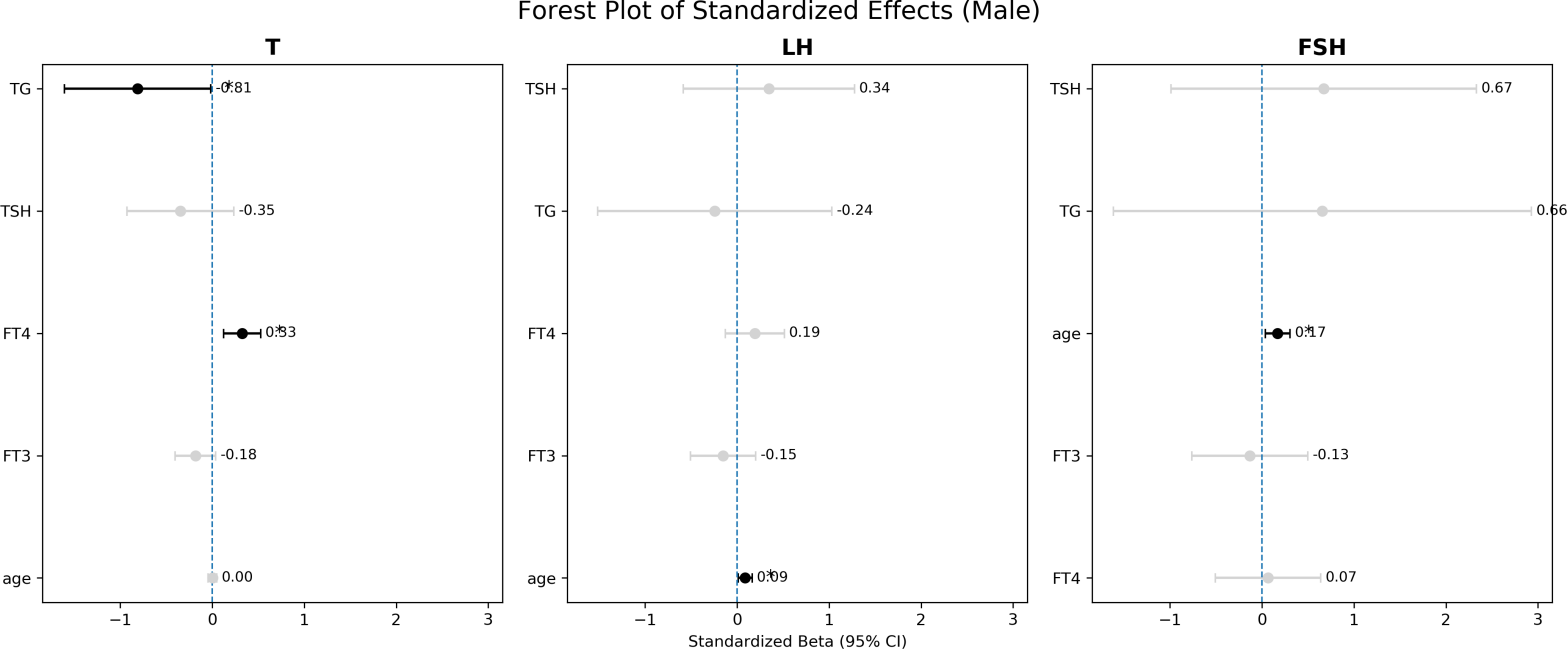
Forest plot adjusted for age, TG and other thyroid function parameters.

To further evaluate the adjusted association between thyroid hormones and sex hormones in men, a multiple linear regression model was constructed. **Figure 3** and **Table 2** for the results. The analysis was conducted along two dimensions: Model A assessed the independent effects of individual thyroid hormones. Model B assessed the combined effects of thyroid hormone sensitivity-derived measures.

**Table 2:** Effects of individual thyroid hormones and the combined effects of thyroid hormone sensitivity-derived indices.

Outcome	Variable	Model A				Model B	
		Model 1 β (p)	Model 1 Std β (p)	Model 2 β (p)	Model 2 Std β (p)	Model B β (p)	Model B Std β (p)
T	FT4	0.236 (0.019)	0.325 (0.019)	0.325 (0.002)	0.448 (0.002)	–	–
	FT3	–	–	-0.182 (0.104)	-0.231 (0.104)	–	–
	TSH	–	–	-0.346 (0.236)	-0.156 (0.236)	–	–
	age	–	–	0.0019 (0.933)	0.012 (0.933)	–	–
	TG	–	–	-0.812 (0.045)	-0.272 (0.045)	–	–
LH	FT3	-0.203 (0.225)	-0.171 (0.225)	-0.151 (0.393)	-0.128 (0.393)	–	–
	FT4	–	–	0.192 (0.234)	0.176 (0.234)	–	–
	TSH	–	–	0.344 (0.459)	0.103 (0.459)	–	–
	age	–	–	0.087 (0.023)	0.341 (0.024)	–	–
	TG		–	-0.243 (0.702)	-0.054 (0.702)	–	–
FSH	FT3	-0.323 (0.277)	-0.154 (0.277)	-0.131 (0.677)	-0.062 (0.677)	–	–
	FT4	–	–	0.065 (0.819)	0.034 (0.819)	–	–
	TSH	–	–	0.671 (0.420)	0.114 (0.420)	–	–
	age	–	–	0.169 (0.014)	0.373 (0.014)	–	–
	TG	–	–	0.656 (0.564)	0.082 (0.564)		
T	FT3_FT4	–	–	–	–	-4.57 (0.054)	-0.283 (0.054)
	TFQI	–	–	–	–	0.707 (0.427)	0.112 (0.427)
	age	–	–	–	–	-0.001 (0.976)	-0.004 (0.976)
	TG	–	–	–	–	-0.693 (0.102)	-0.232 (0.102)

β(p): unstandardized regression coefficient(P); Std β(p): standardized regression coefficient(p).

In the unadjusted analysis of Model A (Model 1), with T as the dependent variable, FT4 was found to be significantly positively correlated with T (Std β = 0.325, P = 0.019). After adjusting for age, TG and other thyroid function parameters, FT4 remained significantly positively correlated with T (Std β 0.448, P = 0.002). FT3, TSH, and age were not statistically significant in the model (P = 0.104, 0.236, and 0.933).

In the regression model for LH and FSH, thyroid hormone levels did not show a consistent significant association before or after adjustment. After full adjustment, age was a significant predictor of elevated LH and FSH levels (LH:Std β=0.341, P = 0.024;FSH:Std β=0.373, P = 0.014).

In Model B, which examines peripheral and central thyroid hormone sensitivity, we included the FT3/FT4 ratio and the TFQI as independent variables and adjusted for age and TG in the analysis. The results showed that the FT3/FT4 ratio exhibited a negative trend with respect to T levels, but this was not statistically significant (P = 0.054). No significant association was observed between TFQI and T (P = 0.427).

Unlike the positive correlation between increased testosterone levels and the T3/T4 ratio observed by Bisschop et al (18) in transgender individuals, the study population in this research consisted of individuals with normal thyroid function who had not received exogenous sex hormone therapy. In addition, the sample size of men in this study was relatively small (n=52), and no analysis by age group was conducted, which may have reduced the ability to detect subtle trends. Therefore, under normal physiological conditions, the FT3/FT4 ratio may have a relatively minor effect on testosterone levels, whereas the absolute FT4 concentration is more strongly associated with androgen levels.

Combining the regression results from Model A and Model B, in the male cohort, compared to derived indicators reflecting thyroid hormone sensitivity or homeostasis (FT3/FT4 and TFQI). The correlation between the absolute concentration of FT4 and T levels is more significant than that of the FT3/FT4 ratio.

### 3.5 Correlations by age group among women and multivariate analysis during childbearing years

**Figure 4:**
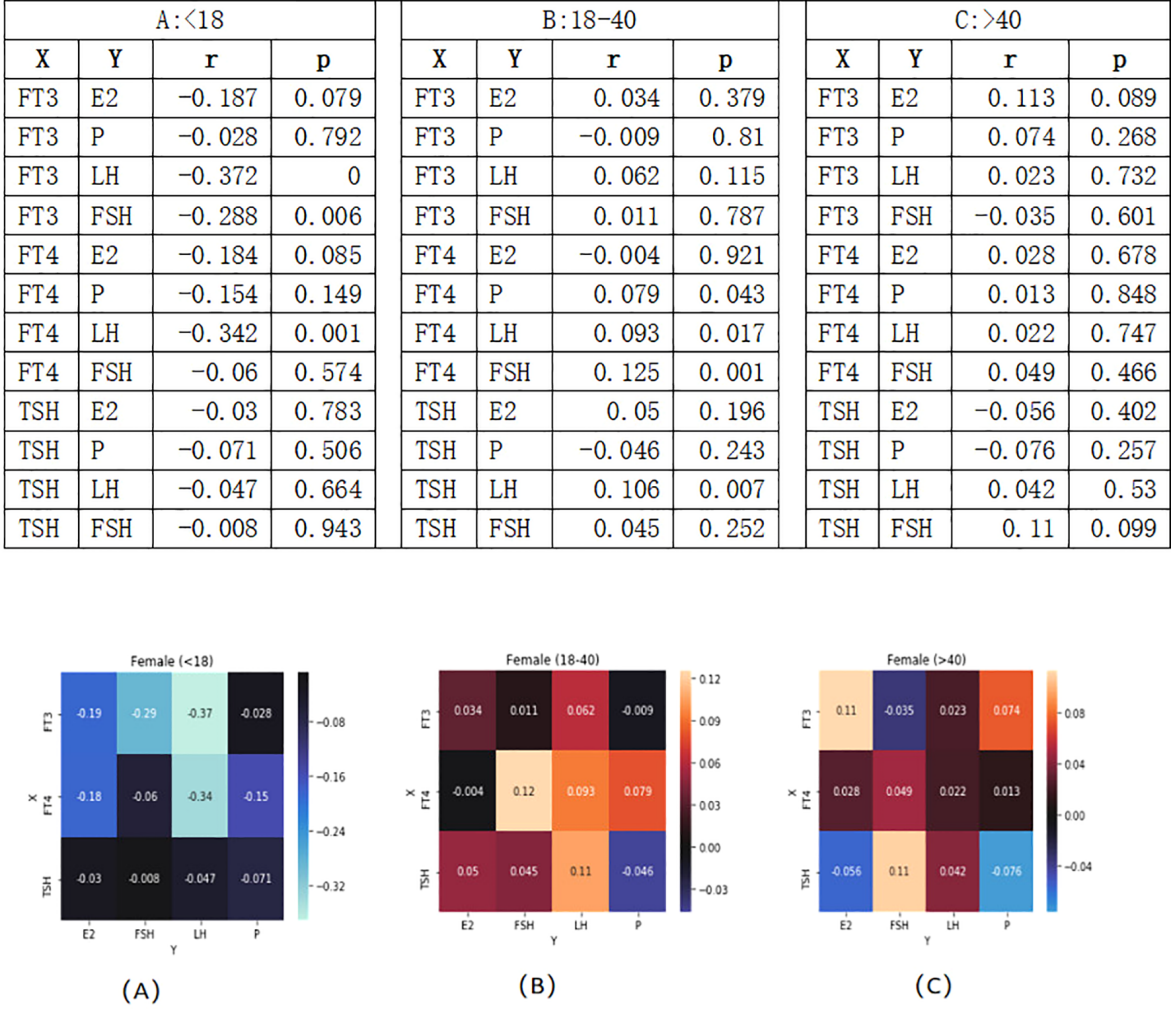
Correlation analysis by age group for women.

Since no significant correlation was observed in the overall female sample, a further correlation analysis was conducted by age group. The results are shown in **Figure 4**.

In the <18-year-old group (**Figure 4A**), FT3 was negatively correlated with LH (r = −0.372, P < 0.001) and FSH (r = −0.288, P = 0.006). FT4 also showed a statistically significant negative correlation with LH levels (r = -0.342, P = 0.001). In the 18–40 age group (**Figure 4B**), FT4 was positively correlated with both LH (r = 0.093, P = 0.017) and FSH (r = 0.125, P = 0.001). FT4 showed a weak positive correlation with P (r = 0.079, P = 0.043). There was also a positive correlation between TSH and LH (r = 0.106, P = 0.007). In the >40-year-old group (**Figure 4C**), no significant correlation was observed between thyroid function markers and reproductive hormones (P > 0.05).

Based on the key associated signals identified in the association analysis, this study constructed a multiple linear regression model in the subcohort of women of childbearing age (18–40 years). The effects on reproductive hormones were evaluated while controlling for potential confounding factors related to age and various components of thyroid function (**Table 3**).

**Table 3:** Multiple regression analysis of thyroid hormones and sex hormones in individuals aged 18–40.

Variable	β	std β	p	Outcome
FT4	0.189428	0.057207856	0.164585569	LH
FT3	0.005788	0.000542034	0.989519965	
TSH	0.709238	0.136014936	0.000431017	
age	-0.242857	-0.177141342	<0.000001	
FT4	0.208472	0.11820376	0.004667379	FSH
FT3	-0.07266	-0.012775047	0.759835568	
TSH	0.148082	0.053317461	0.171242228	
age	0.104464	0.143057511	0.000277446	
FT4	-0.248171	-0.035501656	0.401126333	P
FT3	-0.018864	-0.000836802	0.984257711	
TSH	0.178332	0.016199784	0.681884652	
age	-0.101383	-0.035028348	0.378138945	

The results showed that, with FSH as the dependent variable, FT4 was significantly positively correlated with FSH (Std β = 0.118, P = 0.005), age was also positively correlated with FSH (Std β = 0.143, P < 0.001). No significant association was observed between FT3 and TSH. When LH was the dependent variable, TSH was significantly positively correlated with LH (Std β = 0.136, P < 0.001), and age was negatively correlated with LH (Std β = −0.177, P < 0.001). However, no significant correlations were observed for FT3 or FT4. When P was treated as the dependent variable, no significant associations were observed for FT4, FT3, TSH, or age.

## 4 Discussion

This study found that FT4 levels are positively correlated with testosterone levels in men, whereas FT3 and TSH show no significant association. In the overall female sample, no significant association was found between thyroid hormones and sex hormones; however, when stratified by age, different directions of association were observed across different age groups. Particularly among women of childbearing age, the positive correlations between FT4 and FSH, as well as between TSH and LH, suggest that thyroid hormones may play a role in regulating gonadotropin levels in women. The above results reflect gender differences and age-dependent variations in the thyroid-gonadal axis relationship; an overall analysis may mask weak associations present in specific subgroups.

Even after adjusting for male FT4 and T levels, a positive correlation remained, indicating relatively stable results. FT3, TSH, and the derived ratios FT3/FT4 and TFQI did not show a clear association. These findings suggest that, in the sample studied here, it is FT4 itself—rather than thyroid hormone feedback sensitivity or peripheral conversion characteristics—that is primarily associated with the androgen level. This correlation does not imply causation, nor does it allow us to conclude that FT4 directly determines T levels. Within the normal range of thyroid function, interindividual variations in FT4 may be associated with a certain degree of covariation with male androgen levels. Previous studies suggest that thyroid hormones may contribute to the maintenance of androgen homeostasis by influencing testicular steroid production (19, 20), sex hormone-binding globulin levels (21), and peripheral metabolic processes (22). However, because this study lacks data on SHBG, free testosterone, and more direct indicators of testicular function, the aforementioned mechanisms cannot yet be verified in this study; therefore, they should be regarded as possible explanations rather than definitive conclusions.

Studies of women have found no significant association between thyroid hormones and sex hormones in the overall sample. However, when stratified by age, the different age groups exhibited inconsistent patterns of association. This finding suggests that there may be significant physiological heterogeneity within the female sample, particularly regarding differences in HPG axis status during puberty, childbearing years, and the perimenopausal period. This may influence the statistical relationship between thyroid hormones and gonadotropins. Specifically, in the <18 age group, FT3 and FT4 were negatively correlated with LH/FSH; in the 18–40 age group, the correlation became positive; and in the >40 age group, this correlation was no longer significant. These findings suggest that there may be different patterns of thyroid-gonadal axis interactions across different age groups in women. Analysis by gender and age group among women also revealed that FSH levels in women rise significantly after the age of 40, while FT4 levels remain relatively stable overall. This suggests that as the life cycle progresses, the extent of remodeling in the HPG axis may exceed the changes in the thyroid axis itself.

Among women aged 18–40, FT4 showed a positive correlation with FSH, and TSH showed a positive correlation with LH after adjustment. This result reflects a statistical association; it does not establish a causal relationship (23). On the other hand, the effect sizes of these associations were generally small, and this study did not include key reproductive endocrine data such as menstrual cycle, ovulation status, pregnancy status, and polycystic ovary syndrome (24, 25); therefore, these findings cannot yet be simply extrapolated as direct evidence of follicular development or ovulation regulation. Among women of childbearing age—a relatively homogeneous yet highly complex population—subtle fluctuations within the normal range of thyroid function may be statistically associated with the gonadotropin profile; however, whether this association has clear biological or clinical significance requires further confirmation through prospective studies employing more refined subgroup analyses.

This study also offers some methodological insights. Stratifying by gender and further stratifying by age among women helps identify potential but inconsistent patterns of association. In studies involving interactions within the endocrine axis, population heterogeneity itself is a key factor in interpreting the results. If gender differences, pubertal development, and perimenopausal changes are ignored, certain correlations that exist only in specific subgroups may be weakened in the overall analysis. However, this insight offers value to researchers in terms of study design.

Although this study offers new insights into the relationship between thyroid hormones and sex hormones, it still has certain limitations. First, this study lacks data on BMI and menstrual cycles, and the rate of missing metabolic data among women is high (>80%). Consequently, the multivariate regression analysis for women was unable to adjust for these potential confounding factors. Although TG has been included in the male model to control for metabolic effects, residual confounding may still exist. Since this study is primarily an exploratory analysis, strict corrections for multiple comparisons were not applied to all tests. Therefore, some of the significant results may reflect chance rather than a true effect. To mitigate this risk, we conducted multivariate regression analyses only on indicators that exhibited strong trends and statistical significance, and provided an explanation of the results in the Results and Discussion section. Future studies could further validate these findings using multiple comparison correction methods, supported by larger sample sizes and comprehensive phenotypic data, to confirm their robustness and biological significance.

Second, the gender imbalance in the study sample is due to the fact that the prevalence of thyroid disease and autoimmune thyroid disease is significantly higher in women than in men, making it difficult to obtain a gender-balanced sample in the general population. This means that the study results primarily reflect characteristics of the female population. In particular, the interaction between thyroid hormones and T levels may differ from that with estrogen. Therefore, given these limitations, although the findings of this study suggest a possible gender- and age-related association between thyroid hormones and sex hormones, they are not sufficient to be directly applied to clinical decision-making. Future studies that incorporate menstrual cycle data, metabolic profiles, and longitudinal follow-up information from a more representative population sample will better help to validate the stability of these associations and their potential significance in reproductive endocrine disorders.

Overall, this study indicates that in individuals with normal thyroid function, the relationship between thyroid hormones and sex hormones is not simple or constant, but may be influenced by both gender and age. This suggests that future research on the interaction between the thyroid and gonadal axes should be conducted based on more refined population stratification and a more comprehensive endocrine phenotype.

## 5 Conclusion

This study shows that, in individuals with normal thyroid function, the relationship between thyroid hormones and sex hormones exhibits significant gender differences and is age-dependent. Among men, FT4 levels are positively correlated with testosterone levels after adjustment. No significant associations were observed in the overall female sample; however, different patterns emerged across age groups. Specifically, among women of childbearing age, adjusted correlations were found between FT4 and FSH, as well as between TSH and LH. The above results indicate that, in this study, slight fluctuations in thyroid hormone levels among individuals with normal thyroid function may be associated with reproductive endocrine status; however, it is not possible to infer a causal relationship based on these findings. The specific mechanisms and clinical implications require further clarification through more detailed stratified studies and prospective research.

## Data Availability

The original contributions presented in the study are included in the article/supplementary material. Further inquiries can be directed to the corresponding author.
